# A Sensitive Spectrofluorimetric Method for Curcumin Analysis

**DOI:** 10.1007/s10895-022-02947-w

**Published:** 2022-05-08

**Authors:** Anne Boyina Sravani, Elizabeth Mary Mathew, Vivek Ghate, Shaila A Lewis

**Affiliations:** 1 Department of Pharmaceutics, Manipal College of Pharmaceutical Sciences, Manipal Academy of Higher Education (MAHE), 576104 Manipal, Karnataka India; 2 School of Pharmacy, Faculty of Health Sciences, University of Botswana, Gaborone, Botswana

**Keywords:** Curcumin, Spectrofluorimetric method, HPLC, Validation, Cost-effective, 96 well plates

## Abstract

Curcumin (CUR), a natural polyphenolic compound extracted from the rhizomes of *Curcuma longa*, is used as a pharmaceutical agent, spice in food, and as a dye. Currently, CUR is being investigated for cancer treatment in Phase-II clinical trials. CUR also possesses excellent activities like anti-inflammatory, anti-microbial, and anti-oxidant, therefore quality control is crucial. The present research work was to develop a new, simple, validated and time-saving rapid 96-well plate spectrofluorimetric method for the determination of CUR. The developed method was compared with routinely used high performance liquid chromatography (HPLC) technique. The developed method were found to be linear in the concentration range of 15 to 3900 ng/mL with R^2^ ≥ 0.9983 for spectrofluorimetric and 50-7500 ng/mL with R^2^ ≥ 0.9999 for HPLC method. Accuracy, intraday and interday precision was adequate, with RSD lower than the suggested limits. The limits for the detection and the quantification of CUR were 7 and 15 ng/mL for spectrofluorimetric, and 25 and 50 ng/mL for HPLC respectively. The Bland-Altman analysis demonstrated the similarities between the two methods. The 96-well plate method was successfully applied to determine CUR in solid lipid nanoparticles (SLNs) and chitosan nanoparticles (Chi-NPs). The developed spectrofluorimetric method can hence serve as a possible replacement for the HPLC method for the quantification of CUR in healthcare and food products.

## Introduction

Curcumin (CUR) is a yellow polyphenolic compound extracted from the rhizomes of *Curcuma longa*, commonly known as turmeric. It is widely grown in India and other Asian countries for its use as a spice in cooking, dyes in textiles, food preservative and as a cosmetic agent for skin care.[1] For nutritional purposes, CUR is used in dairy products, beverages, cereals, mustard, food concentrations, sausages, pickles, ice cream, meat, fish, eggs, and bakery products.[2] CUR is also used as a coloring agent in food industries, pharmacy, confectionery, and for dyeing wool, silk, cotton and in combination with natural dyes to get different shades.[3] Rhizomes of *Curcuma longa* are used as expectorants, antiseptics, blood purifiers, insecticides and also used in the treatment of spleen disorders, rheumatism, bronchitis, cough and cold, hypotensive, cholera and syphilis.[4–6] WHO and Food and Agriculture Organization have approved CUR as a food additive.[7, 8] Since many years, CUR has been utilized to treat jaundice, liver ailments and several other medical purposes.[9] CUR has demonstrated chemopreventive properties in several kinds of cancer by suppressing the tumorigenic activity of a wide variety of carcinogens. [10]

Presently, several phase II clinical trials are ongoing on CUR for the chemoprevention of cancer in humans. Clinical trials have indicated that the clinical safety of turmeric (CUR) has no dose-limiting toxicity up to 8–12 g/day in humans.[11–16] Apart from cancer, CUR plays a vital role in treating various diseases which include skin, ulcers, parasitic infections, and auto-immune diseases.[9] Nutraceuticals like CUR can be a promising option as immunity boosters and antidepressants for psychoneuroimmunology (PNI) response.[17] CUR has shown antiviral activity against a broad spectrum of viruses like HIV, HSV-2, HPV viruses, influenza virus, Zika virus, hepatitis virus, adenovirus and is being investigated as a therapeutic option for the management of COVID-19 infection.[18] CUR has the potential to be a promising tool for increasing immunity as well as improving the psychological well-being of COVID infected patients and healthcare workers.[17].

Different analytical methods like UV-Spectrophotometry [19–21], ultra-performance liquid chromatography (UPLC) [22], thin layer chromatography (TLC) [23], high performance liquid chromatography (HPLC) and LC-MS have been reported in literature for the estimation of concentration of CUR in biological as well as pharmaceutical matrices.[24–27] UV-Spectrophotometry is easy to use and gives extremely accurate readings, but it takes time to analyze each sample, and its sensitivity is often inadequate at low sample concentrations. The TLC is a chromatographic technique where compounds can be analyzed based on the polarity of samples. Although it is easy to handle, it is not widely recommended due to its poor resolution. HPLC is extremely quick and efficient with high resolution, accuracy, and high reproducibility. Despite its advantages, HPLC is costly, requires higher quantities of solvents. The UPLC reduces the cost of operation and decreases the consumption of solvent when compared with the HPLC, but the disadvantage is that increase in pressure reduces the life of columns.[28] Chromatography coupled with mass spectroscopy has evolved as a significant analytical technique in sample analysis. Inspite of its sensitivity, the cost and maintenance of the instrument make its usage limited. [29] However, tandem mass spectrometry provides precise structural information about analytes at low concentrations (nano to pictogram/mL).[30–32] Additional limitations of the reported techniques include the inability to quantify lower CUR concentrations, the tedious extraction process, and long sample analysis time.

The spectrofluorimetric method is preferred over other analytical techniques because of its ease of processing, time-saving and cost-efficient nature. CUR emits low quantum yield (< 0.2) fluorescence by absorbing in the visible region. Its emission properties depend highly on the polarity of its environment.[33, 34] The exciting photophysical properties of CUR are responsible for the excited-state intramolecular hydrogen transfer.[35] Recent studies done on the estimation of concentration of CUR using fluorimetry in liposomal formulations and plasma showed that the method is reliable for the detection of CUR.[36] A major challenge in the method is sample processing time, and the volume of solution required for analysis is more.

96-well plates can be easily accommodated in all common instruments like HPLC, GC, LC-MS and can be used for applications such as sample collection and biological assays.[37, 38] The advantages of 96-well plates are that sample processing is easy, requires fewer reagents as the well volume is small and requires fewer plates to run an experiment because more samples can be placed within the plate when compared over conventional sample holders.[39].

**Fig. 1:**
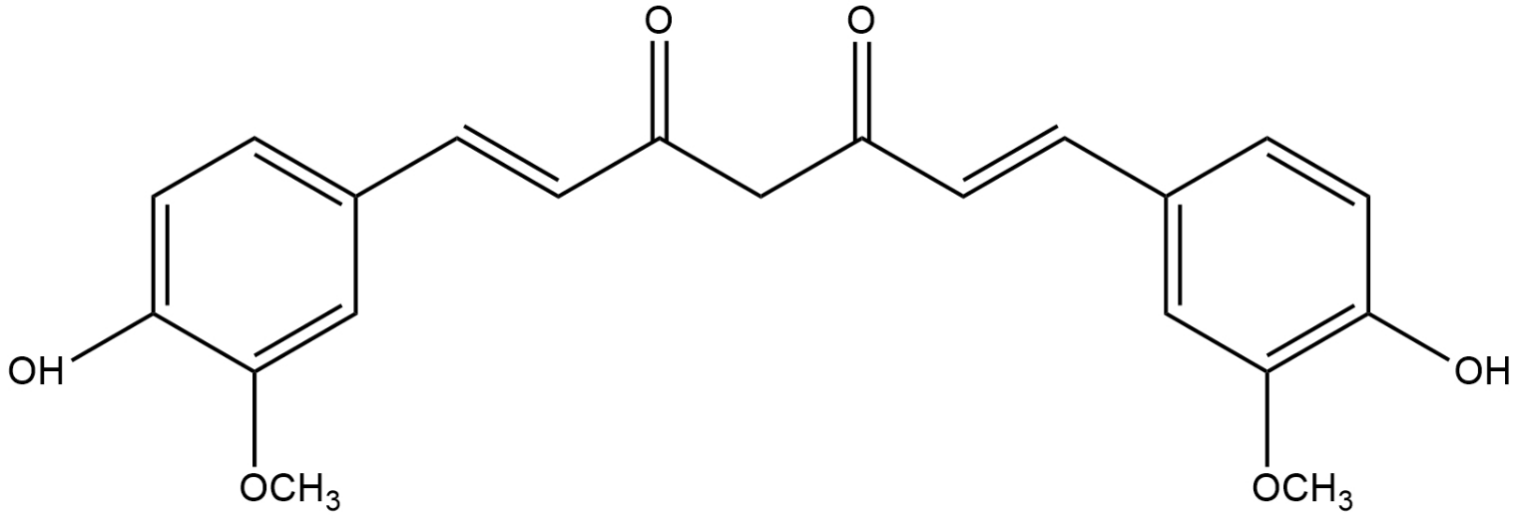
Structure of CUR

The main challenge with CUR is its instability at neutral or basic pH and also its poor absorption when taken orally or applied topically.[40] CUR exists in the forms of diketone and keto-enol and thereby has three acidic protons, two phenolic ones and one enolic proton[41], Fig. 1. Now-a-days, many formulations of CUR such as liposomes, solid lipid nanoparticles (SLNs), chitosan nanoparticles, micelles, phospholipid complexes, hydrogels, powder solutions, solid dispersions and nanoemulsions have been reported to enhance its bioavailability.[42–44] The methods reported in the literature for the estimation of these formulations are UV-spectrophotometry, fluorescence spectrophotometry, HPLC, and LC-MS.[43, 45, 46].

To the best of our knowledge, there is no such spectrofluorimetric method reported for the estimation of concentration of CUR using a 96-well plate. Therefore, there is a need to develop a spectrofluorimetric method for the accurate and precise estimation of CUR in pure form and in formulations. The aim of the present work was to estimate CUR in pure form and formulations by developing a suitable spectrofluorimetric method. ICH guidelines Q2 (R1) guidelines were followed to validate the developed analytical method. [47, 48]

## Materials and Instrumentation

### Materials

CUR was obtained as a gift sample from Arjuna Natural Pvt Ltd, Kerala, India. Sulfobutyl ether-β-CD was gifted by CyDex Pharmaceuticals, USA. Glyceryl Monostearate (GMS) and Chitosan were procured from Sigma-Aldrich, St. Louis. Tween 80 was purchased from Merck Life sciences Pvt Ltd, Mumbai. Poloxamer 407 was procured as gift sample from Signet excipients Pvt Ltd, Mumbai. Acetic acid, ethanol, methanol, dimethyl formamide (DMF), acetone, chloroform, ethyl acetate and acetonitrile and dimethyl sulfoxide (DMSO) were procured from Spectrochem Pvt. Ltd, Mumbai and Finar Ltd, Ahmedabad. All chemicals used in the study were of analytical grade. Purified water used was collected from the Millipore Milli-Q Plus system (Millipore, USA).

### Instrumentation

The fluorescence measurements were performed by using Corning® 96-well flat-bottom plates and a Biotek FLx800 Spectrofluorimetric plate reader. The data was processed on Gen5 Software. The excitation and emission wavelengths for estimation of CUR were set at 485 and 528 nm. [49]. CUR was analyzed using HPLC system with a UV detector (Shimadzu corporation) and a kinetex C18 LC column with particle size 5 μm and dimensions 250 × 4.6 mm. 

## Experimental

### Development of New Method and Optimization

For the estimation of concentration of CUR, a spectrofluorimetric method has been developed using 96-well plates. CUR in the desired concentrations was dissolved in organic solvent. From this, 200 µL of solution was transferred into the well of microplate, where successive serial dilutions were prepared and scanned using spectrofluorimeter microplate reader. The obtained fluorescence intensity (FI) of the sample was subtracted from the blank solvent reading to obtain corrected FI.

The solvents for the development of the spectrofluorimetric method for the detection of CUR were selected based on the compatibility of the plates, the exhibited FI, and the applicable solvent ratios. The solvents ethanol, methanol, DMSO, DMF, chloroform, acetone, ethyl acetate, and acetonitrile were screened to check the FI of CUR. The desired solvents for CUR estimation were selected based on their compatibility with the 96-well plate. Additionally, CUR solutions (100 µg/mL) were prepared in methanol, ethanol, and DMSO and the FI was measured using spectrofluorimeter microplate reader. To determine the effect of solvent ratios on the FI, CUR (100 µg/mL) was analyzed with increased solvent ratios of ethanol and DMSO. FI can vary depending on the sensitivity of the analyzing instrument. The variation in the FI caused by the changes in the instrument sensitivity was recorded by analyzing the solutions at different instrument sensitivity levels (35 and 40).

### HPLC Method

The estimation of concentration of CUR by HPLC method was performed using UV detector at a wavelength of 426 nm. The efficient elution was obtained with Acetonitrile (ACN) and 0.1% Acetic acid (AA) of pH-3.5 in a ratio of 40:60 as the mobile phase. The flow rate and injection volume were set at 1.2 mL/min and 20 µl. The column and oven temperature were maintained at 35°C. The retention time was 17 min.

### Methodology

#### Construction of Calibration Curve for Spectrofluorimetric Method

Stock solution (1000 µg/mL) of CUR was prepared by dissolving 1 mg of CUR in 1mL of DMSO. To prepare working solutions (15–3900 ng/mL), the stock solution was diluted with DMSO in a 96-well plate. The relative fluorescence intensity (RFI) of the solution was measured at 528 nm emission after an excitation at 485 nm against a solvent blank. The obtained fluorescence intensity (FI) of the sample was subtracted from the blank solvent reading to obtain corrected FI. The FI versus concentration of the CUR (ng/mL) was plotted to obtain the calibration graph and corresponding regression equation was derived.

#### Construction of Calibration Curve for HPLC Method

The HPLC method was adopted from the literature with slight modifications.[50] Aliquots of CUR working solutions over the range of 50–7500 ng/mL were prepared in vials using methanol as the solvent. The concentration of CUR (ng/mL) versus area was plotted to get the calibration graph and regression equation.

### Method Validation

The developed analytical method was validated as per ICH guidelines Q2 (R1).[51] It must be noted that all validation parameters for HPLC was followed same as the new method, except for robustness.

#### Specificity

Specificity of spectrofluorimetric and HPLC method was determined by analyzing the blank nanoparticles and CUR loaded nanoparticles in order to assess the possible interference of the formulation excipients in the analysis. [36, 52, 53]

#### Linearity

A range of samples where the analyte concentration was directly proportional to the amount of sample was determined to test the ability of the analytical method.[54] Samples for linearity ranging from 15 to 3900 ng/mL for spectrofluorimetry and 50-7500 ng/mL for HPLC were prepared from stock solution (1000 µg/mL). Triplicates of each standard solution were analyzed using spectrofluorimeter and HPLC. The calibration curve was obtained by plotting the FI versus concentrations for spectrofluorimetry and peak area versus concentration for HPLC. The coefficient of determination (R^2^) was then determined from the calibration curve.

#### Accuracy

The accuracy of the method is the closeness of the measured value to the true value for the sample. [55] The accuracy of the method was determined by the standard addition method. The study was performed at three levels 50%, 100% and 150%. The recovery samples (n = 6) were prepared and then analyzed at their respective wavelengths using spectrofluorimeter and HPLC. The % recovery for pure CUR from the calibration curve was calculated by using following equation:

1.1 $$\% Accuracy=\left(\frac{\text{C}\text{t}}{\text{C}\text{a}}\right)\text{*}100$$

#### Precision

Precision is defined as the closeness of an individual sample subjected to multiple sampling measurements under specified conditions.[56] Reproducibility was also determined through precision studies. Repeatability was determined by preparing six replicates of sample concentrations and measuring their intensity and peak area. An intraday precision study was carried out by preparing a drug solution and analyzing it at two different times in a day. The interday precision was determined after 24 h and the results confirmed adequate reliability. Measurements were reported as the relative standard deviation (%RSD).[57, 58].

#### Robustness

Robustness is the ability of the method to provide unchanged results with deliberate variations. Robustness was examined by evaluating the influence of small variation of method variables, including two different 96-well plates, solvent from two different manufacturers and stability of CUR in the solvent at room temperature for 48 h and the %RSD was calculated.[57–59].

#### Limit of Detection and Limit of Quantification

Limit of detection (LOD) is the lowest amount of analyte in the sample that can be detected by the instrument but not quantified. Limit of quantification (LOQ) is the lowest amount of analyte in the sample that can be detected and quantitatively determined[60].

The LOD and LOQ of the CUR was determined by S/N ratio method. The experiment was performed by measuring signal with low concentrations of analytes of CUR with those of blank. S/N ratio 3:1 and 10:1 was considered acceptable for estimating the detection and quantification respectively.[51].

### Method Comparison Study

Method comparison study was performed to assess the efficiency of newly developed spectrofluorimetric method correlated with HPLC for the estimation of CUR. A total of 60 samples were analyzed using developed spectrofluorimetry as well as HPLC. The collected data was tabulated, and the mean result was calculated. The two means were then compared with each other to determine % standard deviation.[61] The Bland-Altman test was used to assess the comparability of the method using SPSS 16.0. A graph was constructed with the absolute difference between the two paired measurements plotted against their mean value.

### Application of the Method in the Estimation of CUR in Solid Lipid Nanoparticles (SLNs) and Chitosan NPs (Chi-NPs)

CUR loaded SLNs containing glyceryl mono stearate, Tween 80 and Poloxamer 407 were prepared by the microemulsion dilution method.[62, 63] Chi-NPs were prepared using sulfobutyl ether-β-CD as a cross linking agent followed by ionic gelation method.[45, 46, 64] The obtained nanoformulations were characterized with respect to particle size, zeta potential, and polydispersity index (NanoZS, Malvern instruments, UK). To determine the amount of CUR in SLNs and Chi-NPS, 1mL of nanoparticle dispersion was treated with methanol and then centrifuged. The obtained supernatant of SLNs and Chi-NPs was collected and then injected in the HPLC. The amount of CUR was measured from the obtained peak area.

For the spectrofluorimetric method, the SLNs and Chi-NPs were extracted with DMSO, centrifuged and the obtained supernatant was poured into the each well of microplate and then analyzed with spectrofluorimeter. The amount of CUR present in the NPs was calculated from the obtained FI.

### Statistical Analysis

All the trials were conducted in triplicates and the results are presented as mean ± standard deviation. The comparison of the new spectrofluorimetric method and the HPLC was carried out using the Bland-Altman Plot.

## Results and Discussion

CUR exhibited native fluorescence in organic solvents. The aim of the present research is to enhance the FI in order to develop a highly sensitive analytical method for CUR analysis. So, various experimental parameters were investigated including plate compatibility, different solvents, solvent ratio and different sensitivity.

### Selection of Operating Conditions

**Fig. 2:**
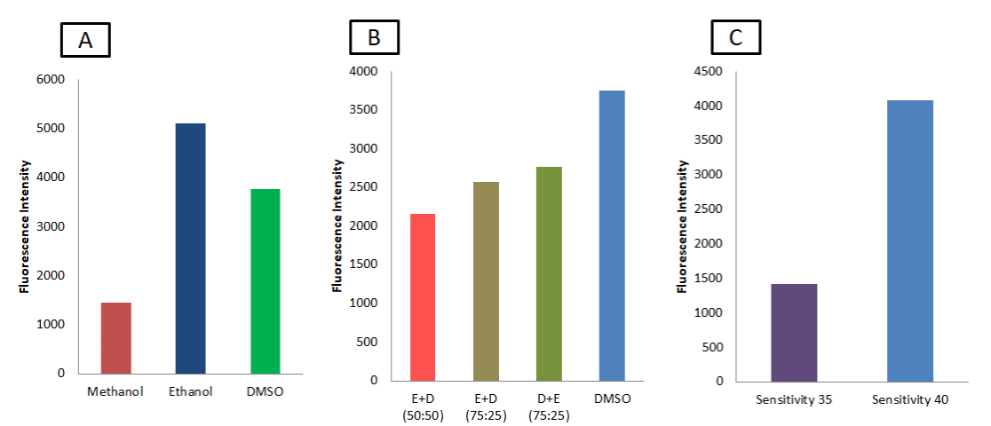
Optimization of Solvents (A) Based on Fluorescence intensity (B) Based on solvent ratios (E – Ethanol and D – DMSO) and (C) Based on effect of detector sensitivity on fluorescence

The desired solvents for CUR estimation were selected based on their compatibility with the 96-well plate. Only methanol, ethanol, and DMSO were found to be compatible with the plate material, whereas the remaining solvents interacted with the plate material, leading to the degradation. Even though CUR ethanolic solutions showed higher FI compared to DMSO and methanol, as shown in Fig. 2A. DMSO was selected as the solvent for analysis due to its higher solubility. The combination of both ethanol and DMSO in different ratios showed an increase in FI with an increased DMSO, but these obtained values were not higher than those obtained with DMSO alone (Fig. 2B). Because CUR dissolved in DMSO could give fluorescence even at low concentrations (below 10 ng/ml), we selected DMSO alone for CUR estimation. Upon dilution with DMSO, the solution was observed under the different sensitivities (35 and 40). At sensitivity 40, the solution exhibited high FI values (Fig. 2C). Hence, we selected sensitivity 40 for further method development.

### Method Validation

#### Validation of the Spectrofluorimetric Method

**Fig. 3:**
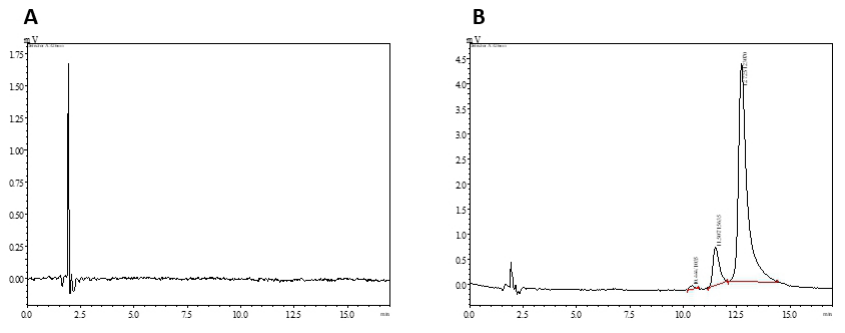
Representative chromatograms profiles obtained for (A) Blank nanoparticles (B) CUR loaded nanoparticles

**Fig. 4:**
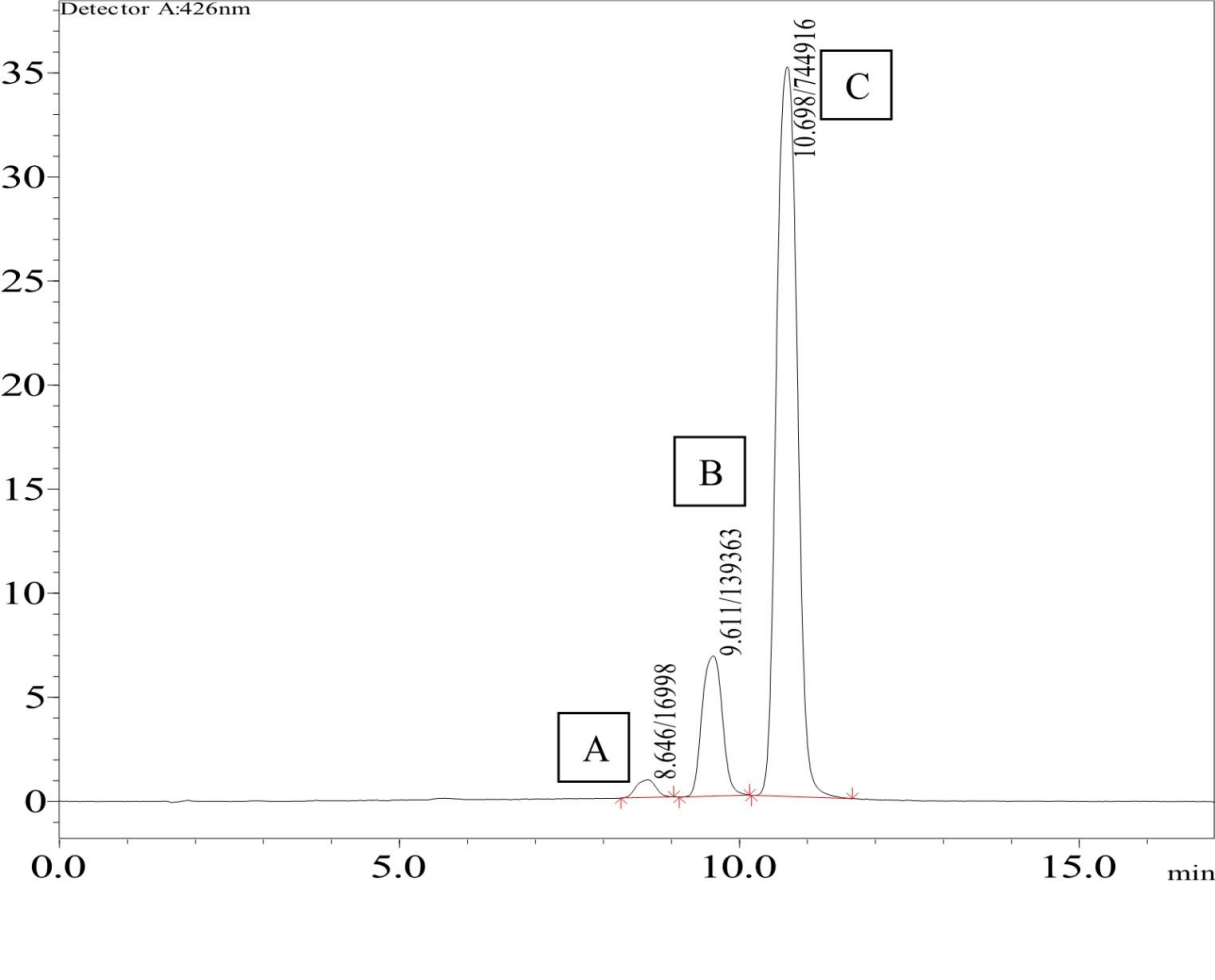
HPLC chromatogram of CUR (A) bismethoxycurcumin, (B) desmethoxycurcumin and (C) curcumin

Validation of the spectrofluorimetric method was done for analytical parameters under optimized conditions. For specificity, there was no fluorescence intensity shown for blank nanoparticles with spectrofluorimetric method. [36] Specificity for HPLC method was determined by comparing the chromatograms obtained for blank nanoparticles and CUR loaded nanoparticles. The chromatogram (Fig. 3), confirms that the excipients in the nanoparticles did not interfere in the CUR peak obtained for CUR loaded nanoparticles, evidencing the specificity of the method. [53] In a previous study, the linearity concentration range for estimation of CUR using spectrofluorimetric analysis was 50-500ng/mL with %accuracy of 80, 100 and 120% showed percent recoveries ranging from 96.33 to 100.75%. The precision results from the literature showed an RSD of less than 8% which was more than the present developed method of < 5% [36]. The results obtained from the regression analysis of present developed spectrofluorimetric method showed linearity over the concentration range of 15–3900 ng/mL and its R^2^ was 0.9983, as shown in Table 1; Fig. 4. The percentage accuracy for the different concentration levels (50, 100 and 150%) was found to be 94.17, 103.16 and 99.81%, confirming the accuracy of the suggested method for the estimation of CUR. The results are tabulated in Table 2. Precision was determined by assessing intra-day and inter-day relative standard deviation. Results shown in Table 1indicated that inter-day and intra-day variability is reasonable and %RSD values were lower than 5% which were in acceptable range.[36] The robustness of spectrofluorimetric method determined using two different 96-well plates, solvent from two different manufacturers and stability of CUR in solvent showed a %RSD of 2.874–4.957%, 1.449–2.991% and 1.702–2.662% respectively. [57, 58] The LOD and LOQ for developed spectrofluorimetric method were found to be 7 ng/mL and 15 ng/mL and for HPLC it was found to be 25 ng/mL and 50 ng/mL respectively.

**Table 1:** Analytical parameters of the developed spectrofluorimetric and HPLC methods

Method	Linear equation	Linear range	R^2^	LOD	LOQ	Intra-day RSD (%) (n = 3)	Inter-day RSD (%) (n = 3)
Spectro-fluorimetry	Y = 71.781x + 2.8617	Y = 15–3900 ng/mL	0.9983	7 ng/mL	15 ng/mL	4.763	4.645
HPLC	Y = 100.71x – 1984	50-7500 ng/mL	0.9999	25 ng/mL	50 ng/mL	0.305	0.799

**Table 2:** Accuracy results of different levels of CUR using Spectrofluorimetry and HPLC

Levels	Spectrofluorimetry		HPLC	
	**Recovery (%)**	**RSD (%)**	**Recovery (%)**	**RSD (%)**
**50%**	94.17	5.6	97.06	1.04
**100%**	103.16	12.84	95.92	0.57
**150%**	99.81	9.4	97.88	3.15

#### Validation of the HPLC method

The chromatogram exhibited three peaks, with the major peak referred to as CUR and two minor peaks identified as demothoxycurcumin and bismethoxy curcumin, which are shown in Fig. 4. [65]

**Fig. 5:**
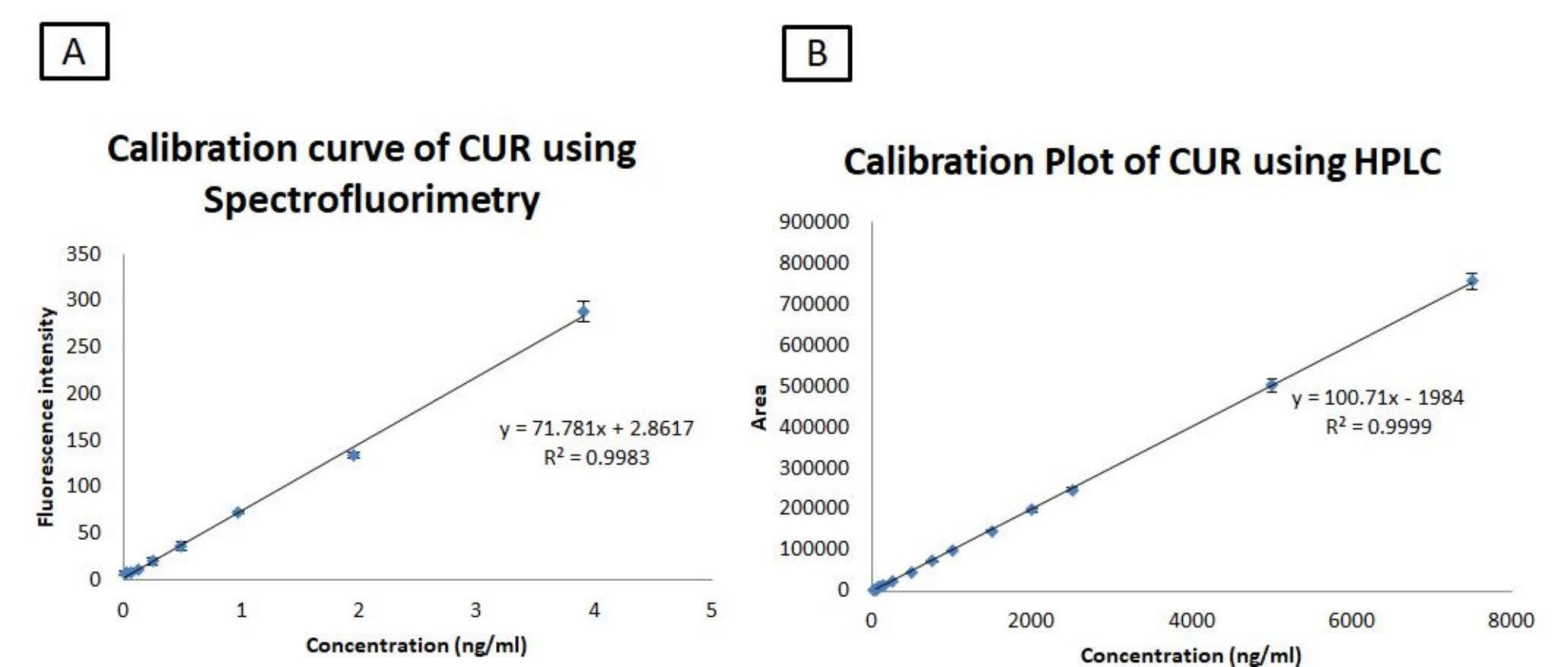
Calibration curve of CUR using Spectrofluorimetric (A) and HPLC method (B)

The developed HPLC method was found to be linear over the range of 50-7500 ng/mL. The calibration curve plotted was found to be linear with a regression of 0.9999 shown in Fig. 5. Intra-day and inter-day precision results for the estimation of CUR are shown in Table 1. The developed method was found to be precise, as intra-day and inter-day batches showed RSD < 2% for HPLC and < 5% for spectrofluorimetric method. The percentage accuracy ranged from 97.06 to 97.88% with an RSD of less than 4% shown in Table 2, indicating a low variability and close agreement between the experimental and theoretical concentration values.[53] LOD and LOQ values for the CUR were found to be 25 ng/mL and 50 ng/mL.

### Method Comparison Studies

The comparability of the method was assessed statistically by the Bland-Altman test using SPSS 16.0.[61] The Bland-Altman plot gives the difference between a pair of measurements made with the two methods with respect to the mean of this pair of measurements. The values which fall within the “limits of agreement” summarize how well the two methods of measurements matched. If the two methods provide similar results, then the difference between them will be minimal, with an average nearly zero, and the limits of agreement will be zero.[66].

The differences between spectrofluorimetric and HPLC method were plotted against the mean of the two measurements. Any possible relation between measurement error and true value can be evaluated by plotting differences against mean.

**Fig. 6:**
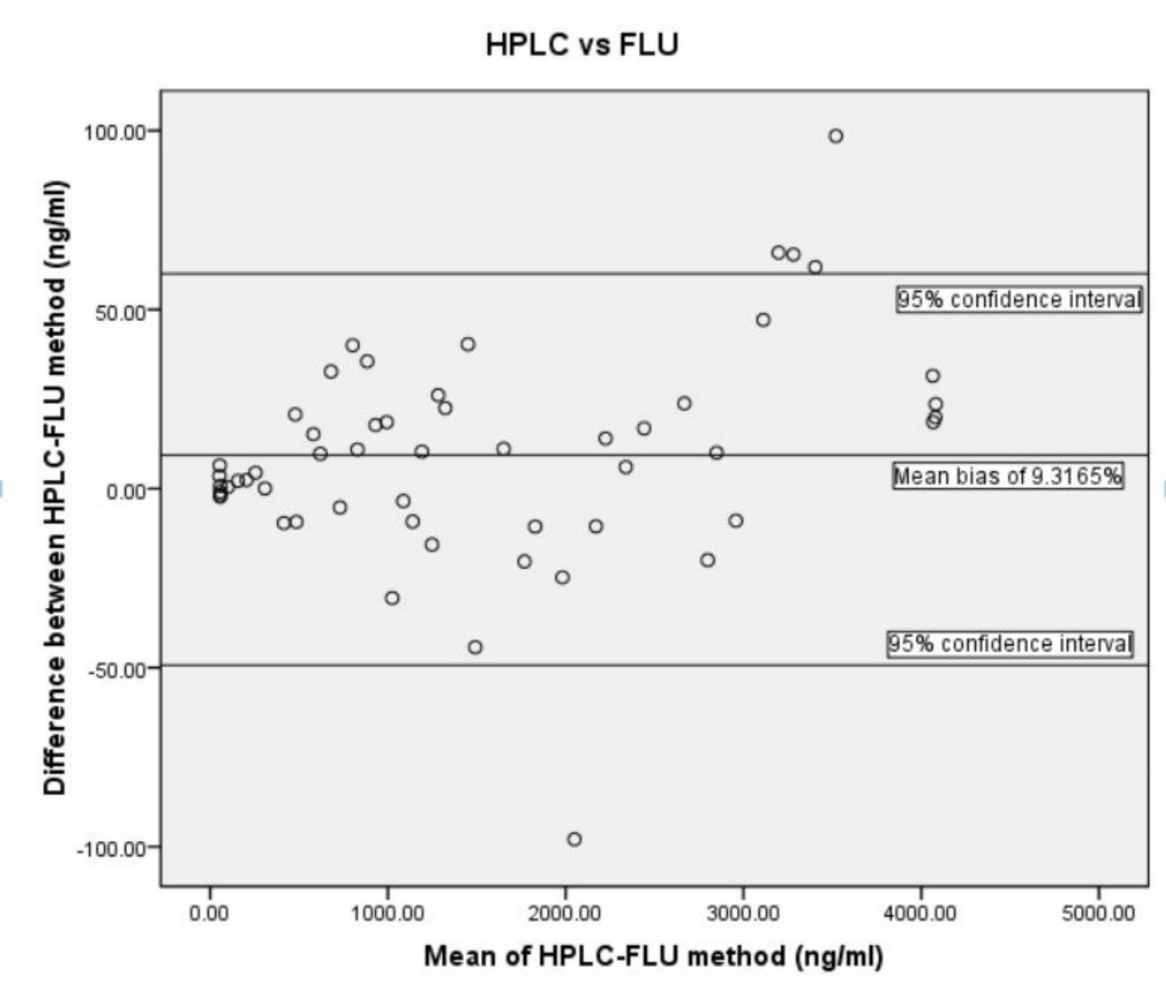
Bland-Altman plot of HPLC and FLU method

The plotted graph in Fig. 5 showed mean ± SD of 1588.9 ± 1279.75 ng/mL. The average of mean difference is 9.316 units which is showed as a center line in Fig. 6. As the mean difference is not zero, it indicates that the spectrofluorimetric method measures 9.316 units more than HPLC. The agreement limits are from − 49.3 to 67.5 ng/mL. This clearly demonstrates the potential of using HPLC and FLU method for the estimation of CUR.

**4.4 Application of the method in the estimation of CUR in Solid lipid nanoparticles (SLNs) and Chitosan NPs (Chi-NPs)**.

The developed spectrofluorimetric and HPLC method was applied for the determination of CUR in Chi-NPs and SLNs. Spectrofluorimetric analysis was carried out in 96-well plates with clear bottom. Particle size and zeta potential for SLNs were found to be 542.40 nm and + 53.50 mV respectively, while Chi-NPs were 537.10 nm and − 17.70 mV. The amount of CUR found in Chi-NPs and SLNs was 2.12 ± 0.182 mg/mL and 2.48 ± 0.12 mg/mL by the spectrofluorimetric method and 3.36 ± 0.227 mg/mL and 3.17 ± 0.141 mg/mL by HPLC method. The results obtained from both the methods are nearly matching with each other, which indicate that the spectrofluorimetric method can be used for the estimation of CUR.

Spectrofluorimetric method is an attractive option for the pharmaceutical analysis and it can also be used for food quality analysis because of its less time for sample preparation, quick analysis, no complex procedure and no much manpower required.[67].

## Conclusions

The proposed spectrofluorimetric method provides an inexpensive, rapid, specific, sensitive, precise, reliable and accurate method for the analysis of CUR using spectrofluorimeter plate reader. The high sensitivity and analysis speeds are the substantial advantages of this method when compared with other methods. This systematically developed method meets all criteria required as per ICH guidelines. This work also describes a validated spectrofluorimetric method for the determination of the CUR. Hence, it may be applied for the routine analysis of CUR in pure form and nanoformulations. Hence, the spectrofluorimetric method can serve as a viable alternative method to replace the reported methods and high cost HPLC methods.

## Data Availability

Not Applicable.

## References

[1] Jayaprakasha GK, Jena BS, Negi PS, Sakariah KK (2002). Evaluation of Antioxidant Activities and Antimutagenicity of Turmeric Oil: A Byproduct from Curcumin Production. Zeitchrift fur naturforchung C.

[2] Sharifi-Rad J, El Rayess Y, Abi Rizk A, Sadaka C, Zgheib R, Zam W, Sestito S, Rapposelli S, Neffe-Skocińska K, Zielińska DSB (2020). Turmeric and its major compound curcumin on health: bioactive effects and safety profiles for food, pharmaceutical, biotechnological and medicinal applications. Front Pharmacol.

[3] Lawhavinit O, Sincharoenpokai P, Sunthornandh P (2011). Effects of Ethanol Tumeric (Curcuma longa Linn.) Extract Against Shrimp Pathogenic Vibrio spp. and on Growth Performance and Immune Status of White Shrimp (Litopenaeus vannamei). Agric Nat Resour.

[4] Prof DP, Gupta C (2014) Phytochemicals of nutraceutical importance from Curcuma longa L and their role in human health.Phytochem Nutraceutical Importance266–287

[5] Nahak GSR (2011). Evaluation of antioxidant activity in ethanolic extracts of five curcuma species. Int Res J Pharm.

[6] Yu ZF, Kong LD, Chen Y (2002). Antidepressant acti v ity of aqueous extracts of Curcuma longa in mice. J Ethnopharmacol.

[7] Heath DD, Pruitt MA, Brenner DE, Rock CL (2003). Curcumin in plasma and urine: quantitation by high-performance liquid chromatography. J Chromatogr.

[8] Anand P, Kunnumakkara AB, Newman RA, Aggarwal BB (2007). Bioavailability of Curcumin: Problems and Promises. Mol Pharm.

[9] Amalraj A, Pius A, Gopi S, Gopi S (2016). Biological activities of curcuminoids, other biomolecules from turmeric and their derivatives e A review. J Tradit Chinese Med Sci.

[10] Maheshwari RK, Singh AK, Gaddipati J, Srimal RC (2006). Multiple biological activities of curcumin: A short review. Life Sci.

[11] Farghadani RNR (2021). A " Window Trial " on Curcumin for Invasive Breast Cancer Primary Tumors. Cancers (Basel).

[12] Tuyaerts S, Rombauts K, Everaert T, Van Nuffel AMAF (2019). A Phase 2 Study to Assess the Immunomodulatory Capacity of a Lecithin-based Delivery System of Curcumin in Endometrial Cancer. Front Nutr.

[13] Ferri C, West K, Otero K, Kim YH (2018). Effectiveness of Curcumin for Treating Cancer During Chemotherapy. Altern Complemenatry Ther.

[14] Passildas-Jahanmohan J, Eymard JC, Pouget M, Kwiatkowski F, Van Praagh I, Savareux L, Atger M, Durando X, Abrial C, Richard DGCA (2021). Multicenter randomized phase II study comparing docetaxel plus curcumin versus docetaxel plus placebo in first‐line treatment of metastatic castration‐resistant prostate cancer. Cancer Med.

[15] Li YH, Niu YB, Sun Y, Zhang F, Liu CX, Fan LMQ (2015). Phase II A Trial of Curcumin Among Patients With Prevalent Subclinical Neoplastic Lesions (Aberrant Crypt Foci). World J Gastroenterol.

[16] Miller AH, Miller AH (2017) Phase II Study of Curcumin vs Placebo for Chemotherapy-Treated Breast Cancer Patients Undergoing Radiotherapy. Clin Trials gov Identifier NCT01740323

[17] Soni VK, Mehta A, Shukla D, Kumar SVN (2020). Fight COVID-19 depression with immunity booster: Curcumin for psychoneuroimmunomodulation. Asian J Psychiatr.

[18] Zahedipour F, Atefe S, Thozhukat H (2020). Potential effects of curcumin in the treatment of COVID-19 infection. Phyther Res.

[19] Singh A, Avupati VR (2017). Development and Validation of UV-Spectrophotometric method for the Estimation of Curcumin in Standardised Polyherbal Formulations. J Young Pharm.

[20] Sharma K, Agrawal SS, Gupta M (2017) Development and Validation of UV spectrophotometric method for the estimation of Curcumin in Bulk Drug and Pharmaceutical Dosage.Int J Drug Deliv4

[21] Kadam PV, Bhingare CL, Nikam RY (2014). Development and validation of UV Spectrophotometric method for the estimation of Curcumin in cream formulation. Pharm Methods.

[22] Cheng J, Weijun K, Yun L (2010). Development and validation of UPLC method for quality control of Curcuma longa Linn.: Fast simultaneous quantitation of three curcuminoids. J Pharm Biomed Anal.

[23] Zhang JS, Guan J, Yang FQ (2008). Qualitative and quantitative analysis of four species of Curcuma rhizomes using twice development thin layer chromatography. J Pharm Biomed Anal.

[24] Wiley J (2009). A rapid and simple HPLC method for the determination of curcumin in rat plasma: assay development, validation and application to a pharmacokinetic study of curcumin liposome. Biomed Chromatogr.

[25] Jangle RD, Thorat BN (2013). Reversed-phase high-performance liquid chromatography method for analysis of curcuminoids and curcuminoid-loaded liposome formulation. Indian J Pharm Sci.

[26] Agan LIJ, Ao MOR, Akariah KUKS (2002). Improved HPLC Method for the Determination of Curcumin, Demethoxycurcumin, and Bisdemethoxycurcumin. J Agric Food Chem.

[27] Wichitnithad W, Jongaroonngamsang N, Rojsitthisak P (2009). A Simple Isocratic HPLC Method for the Simultaneous Determination of Curcuminoids. Phytochem Anal.

[28] Taleuzzaman M, Ali S, Sj G, Ss I (2015). Ultra Performance Liquid Chromatography (UPLC) - A Review. Austin J Anal Pharm Chem.

[29] Kotha RR, Luthria DL (2019). Curcumin, Biological, Pharmaceutical, Nutraceutical and Analytical Aspects. Molecules.

[30] Carolina Alves R, Perosa Fernandes R, Fonseca-Santos B, Damiani Victorelli FCM (2019). A Critical review of the properties and anlaytical methods for the determination of curcumin in biological and pharmaceutical matrices. Crit Rev Anal Chem.

[31] Li W, Yang H, Buckley B (2019). A Novel Triple Stage Ion Trap MS method validated for curcumin pharmacokinetics application: a comparison summary of the latest validated curcumin LC/MS methods. J Pharm Biomed Anal.

[32] Huang Y, Adeleye AS, Zhao L, Minakova AS (2019). Antioxidant response of cucumber (Cucumis sativus) exposed to nano copper pesticide: Quantitative determination via LC-MS / MS. Food Chem.

[33] Patra D, Malaeb NN (2012). Fluorescence modulation of 1,7-bis(4-hydroxy-3-methoxyphenyl)-1,6-heptadiene-3,5-dione by silver nanoparticles and its possible analytical application. Luminescence.

[34] Priyadarsini KI (2009). Photophysics, photochemistry and photobiology of curcumin : Studies from organic solutions, bio-mimetics and living cells. J Photocheistry Photobiol C Photochem Rev.

[35] Mouslmani M, Patra D (2014). Revoking excited state intra-molecular hydrogen transfer by size dependent tailor-made hierarchically ordered nanocapsules. RSC Adv.

[36] Karimi M, Mashreghi M, Saremi SS, Jaafari MR (2020). Spectrofluorometric Method Development and Validation for the Determination of Curcumin in Nanoliposomes and Plasma. J Fluoresc.

[37] Qian Z, Le J, Chen X (2017). High-throughput LC – MS / MS method with 96-well plate precipitation for the determination of arotinolol and amlodipine in a small volume of rat plasma: application to a pharmacokinetic interaction study. J Sep Sci.

[38] Royle L, Campbell MP, Radcliffe CM (2008). HPLC-based analysis of serum N-glycans on a 96-well plate platform with dedicated database software. Anal Biochem.

[39] Auld DS, Ph D, Coassin PA et al (2020) Microplate Selection and Recommended Practices in High-throughput Screening and Quantitative Biology Introduction to Microplates in High-Throughput Screening and Quantitative Biology.Assay Guid Man1–50

[40] Gupta NK, Nahata A, Dixit VK (2016). Development of Spectrofluorimetric Method for the determination of Development of a spectrofluorimetric method for the determination of curcumin. Asian J Trad Med.

[41] Tsaplev YB, Lapina VA, Trofimov AV (2020). Curcumin in dimethyl sulfoxide: Stability, spectral, luminescent and acid-base properties. Dye Pigment.

[42] Prasad S, Aggarwal BB (2014). Recent Developments in Delivery, Bioavailability, Absorption and Metabolism of Curcumin : the Golden Pigment from Golden Spice. Cancer Res Treat Off J Korean Cancer Assoc.

[43] Dutta AK, Ikiki E (2013). Novel Drug Delivery Systems to Improve Bioavailability of Curcumin Bioequivalence & Bioavailability Novel Drug Delivery Systems to Improve Bioavailability of Curcumin. J Bioequivalence Bioavailab.

[44] Im K, Maliakel A, Gopakumar G (2015). Improved blood – brain-barrier permeability and tissue distribution following the oral administration of a food-grade formulation of curcumin with fenugreek fibre. J Funct Foods.

[45] Khan A, Mehdi SH, Ahmad I, Rizvi MMA (2016). Characterization and anti-proliferative activity of curcumin loaded chitosan nanoparticles in cervical cancer. Int J Biol Macromol.

[46] Nair RS, Morris A, Billa N, Leong C (2019). An Evaluation of Curcumin-Encapsulated Chitosan Nanoparticles for Transdermal Delivery. AAPS PharmSciTech.

[47] Rapalli VK, Kaul V, Gorantla S (2020). UV Spectrophotometric method for characterization of curcumin loaded nanostructured lipid nanocarriers in simulated conditions: Method development, in-vitro and ex-vivo applications in topical delivery. Spectrochim Acta Part A Mol Biomol Spectrosc.

[48] Singhvi G, Kalantare P, Harish D, Saha RN (2013). Spectrophotometric determination of nor-epinephrine serotonin reuptake inhibitor (SNRI) drug Milnacipran in pure and in dosage forms. Asian J Chem.

[49] Mogharbel BF, Francisco C, Carolina A (2018). Fluorescence properties of curcumin-loaded nanoparticles for cell tracking. Int J Nanomedicine.

[50] Thomas A, Radha A, Ragavendran P, EFFECTIVE HPLC METHOD FOR THE ANALYSIS OF CURCUMINOIDS (2016). A COST. Hygeia JD Med.

[51] DE LT VM, SINGH RP, DERENDORF H (2010). ICH Q2 (R1): Validation of Analytical Procedures: Text and Methodology, 1996 ICH Q2 (R1): Validation of Analytical Procedures: Text and Methodology, 1996, 1996. J Chromatogr B Analyt Technol Biomed Life Sci.

[52] Mazzarino L, Bellettini IC, Minatti E, Lemos-Senna E (2010). Development and validation of a fluorimetric method to determine curcumin in lipid and polymeric nanocapsule suspensions. Brazilian J Pharm Sci.

[53] Vieira ES, Lemos-senna E (2020). Application of a New Validated HPLC-PDA Method for Simultaneous Determination of Curcumin and Melatonin in Hyaluronic Acid-Coated Nanoemulsions. J Braz Chem Soc.

[54] Baldi A, Panwar MS, Swapnil G (2015). Development and validation of spectrophotometric method for determination of ropinirole hydrochloride in bulk and formulation. Inven Rapid Pharm Anal Qual Assur.

[55] Baldi A, Patidar AK, SanadyA J (2010). Method Development and Validation for Estimation of Quetiapine Fumarate by RP-HPLC. Asian J Res Chem.

[56] Warule Pooja SPVP, GSA (2017). Development and Validation of UV Spectrophotometric Method for the Estimation of Curcumin in an Ayurvedic Formulation Haridrakhand. Int J Pharm Drug Anal.

[57] Bento D, Borchard G, Gonçalves T, Borges O (2013). Validation of a New 96-Well Plate Spectrophotometric Method for the Quantification of Compound 48 / 80 Associated with Particles. AAPS PharmSciTech.

[58] Taverniers I, Loose M, Van De, Bockstaele E (2004). Trends in qualirty in the analytical laboratory. II. Analytical method validation and quality assurance. Trends Anal Chem.

[59] Oriquat G, Osman A, Abdul-Azim M, Abuhamdah S (2014). Development and validation of a stability indicating spectrofluorimetric method for the determination of lanzoprazole via its degradation product. J Appl Pharm Sci.

[60] Wenzl T, Haedrich J, Schaechtele A, Robouch PSJ (2016) Guidance Document on the Estimation of LOD and LOQ for Measurements in the Field of Contaminants in Feed and Food. Publ Off Eur Union Luxembg 58

[61] Mazzo DJCM (1992). Analytical Method Comparison Based upon Statistical Power Calculations. Pharm Res.

[62] Kursad A, Yenilmez A, Eroglu H (2017). Evaluation of radiolabeled curcumin-loaded solid lipid nanoparticles usage as an imaging agent in liver-spleen scintigraphy. Mater Sci Eng C.

[63] Kakkar V, Muppu SK, Chopra K, Kaur IP (2013) Curcumin loaded solid lipid nanoparticles: An efficient formulation approach for cerebral ischemic reperfusion injury in rats. Eur J Pharm Biopharm 1–8. 10.1016/j.ejpb.2013.02.005 10.1016/j.ejpb.2013.02.005 23454202

[64] Mehdi SH, Ahmad I (2018). Physicochemical Characterization of Curcumin Loaded Chitosan Nanoparticles: Implications in Cervical Cancer. Anticancer Agents Med Chem.

[65] Rodero CF, Maria G, Calixto F (2018). Curcumin-loaded liquid-crystalline systems for controlled drug release and improved treatment of vulvovaginal candidiasis. Mol Pharm.

[66] Jordan H, Drummond G (2016). Reporting of method comparison studies: a review of advice, an assessment of current practice, and specific suggestions for future reports. Br J Anaesth.

[67] Manuel FJ (2019) Fluorescence: Food Applications ☆ Fluorescence: Food Applications, 3rd edn. Elsevier Inc

